# Radiotherapy, Phototherapy, Radium and the High Frequency Currents

**Published:** 1904-11

**Authors:** 


RADIOTHERAPY, PHOTOTHERAPY, RADIUM AND THE HIGH FREQUENCY CURRENTS. By Chas. Warrenne Allen, M. D. D. Appleton & Co. N. Y.

The growth in the use of physical therapeutics has occasioned much stimulation to the making of books on the subject. In

Allen's Work one will find a good guide for carrying out the various methods by radiotherapy, phototherapy and the like. There is a sufficiently ample consideration of the physics of these agents and of their necessary apparatus. The book is well illustrated and interlarded with illustrative cases—a helpful addition. As these modes of combatting disease are still in a measure *sub judice* the publication of ever-accumulating evidence is desirable. Dr. Allen has written his book in a judicial spirit rather than from the standpoint of an enthusiast. This, of course, adds to the merit of a very meritorious commendable work.

